# Supplementary material for: Computational Analysis of Triazole-Based Kojic Acid Analogs as Tyrosinase Inhibitors by Molecular Dynamics and Free Energy Calculations
Source: Molecules. 2022 Nov 23;27(23):8141. doi: 10.3390/molecules27238141 (PMC9735930; doi:10.3390/molecules27238141)
Supplement: Supplementary file 1 [file molecules-27-08141-s001.zip › molecules-2045287-supplementary materials.pdf]

Computational Analysis of Triazole-based Kojic Acid Analogs as Tyrosinase Inhibitors by Molecular Dynamics and Free Energy Calculations

Lucas Sousa Martins, Reinaldo W. A. Gonçalves, Joana J. S. Moraes, Cláudio Nahum Alves and José Rogério A. Silva

Supplementary Materials

Figure S1. Regression plot between MOLDOCK scoring (Kcal/mol) and IC50 (μM) for all TYR systems.

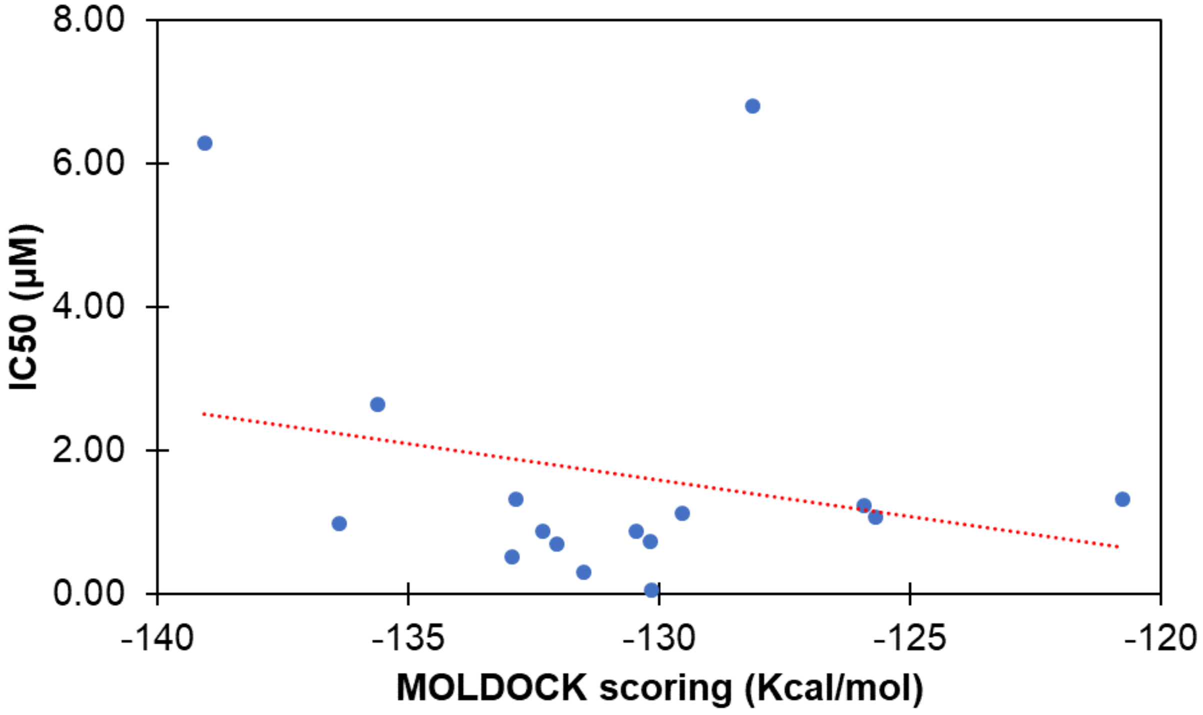

Figure S2. Plot of MD time (ps) versus RMSD (Å) for all TYR systems.

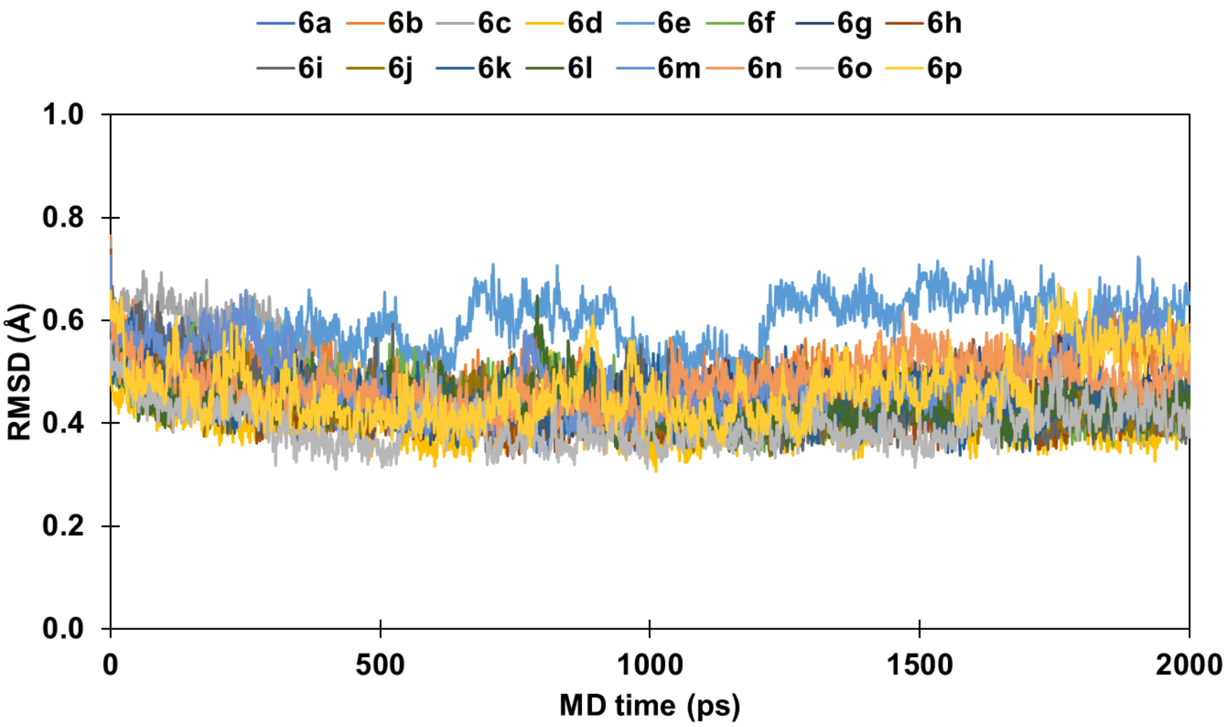

Table S1. LIE empirical parameters used for TYR systems.

Inhibitor	α	β	γ
6a	0.18	0.37	17.33
6b	0.18	0.37	17.33
6c	0.18	0.37	17.33
6e	0.18	0.37	17.33
6f	0.18	0.37	17.33
6h	0.18	0.33	17.33
6i	0.18	0.37	17.33
6j	0.18	0.35	17.33
6l	0.18	0.37	17.33
6m	0.18	0.37	17.33
6n	0.18	0.37	17.33
6o	0.18	0.37	17.33
6p	0.18	0.37	17.33

Table S2. vdW interactions (in Kcal/mol) computed for all TYR systems.

Residue	6a	6b	6c	6e	6f	6h	6i	6j	6l	6m	6n	6o	6p
**Phe197**	-1.97	-1.98	-2.43	-2.1	-1.32	-3.13	-4.19	-3.79	-3.38	-3.02	-2.99	-2.34	-2.72
**Gly200**	-1.14	-0.53	-0.8	-1.28	-0.43	-1.78	-2.05	-2.33	-1.47	-1.77	-1.49	-1.62	-1.93
**Pro201**	-3.06	-2.25	-2.92	-3.48	-1.32	-3.50	-3.61	-3.89	-3.37	-4.14	-3.51	-4.04	-4.08
**His204**	-0.92	-0.93	-0.77	0.21	-0.72	-0.75	-1.14	-1.09	-0.10	-1.18	-0.54	-0.61	-0.41
**Asn205**	-4.35	-3.96	-5.14	-5.60	-2.89	-5.37	-5.10	-5.44	-5.56	-5.13	-5.43	-5.27	-5.66
**Arg209**	-3.33	-3.31	-3.13	-3.78	-3.82	-3.18	-3.42	-3.72	-2.99	-3.90	-3.30	-3.66	-3.08
**Met215**	-1.00	-0.78	-0.77	-1.06	-1.33	-0.80	-0.49	-0.79	-1.08	-0.74	-0.60	-1.36	-1.24
**Gly216**	-1.5	-1.51	-1.48	-1.63	-1.53	-1.38	-1.33	-1.65	-1.54	-1.40	-0.97	-1.48	-1.61
**Val217**	-1.78	-1.54	-1.81	-1.62	-2.24	-1.31	-1.52	-1.60	-1.67	-1.09	-1.04	-1.69	-1.69
**Val218**	-2.27	-2.28	-2.30	-3.79	-3.08	-2.16	-3.06	-2.91	-3.64	-1.88	-2.24	-3.64	-3.28
**His231**	-1.02	-1.13	-1.10	-0.97	-1.23	-1.15	-0.75	-1.06	-1.04	-1.16	-1.17	-1.37	-1.38
**Cu2(A)**	-0.91	-0.96	-0.76	-0.41	-0.64	-0.81	-4.90	-0.61	-0.43	-0.96	-0.73	-0.59	-0.60
**Cu2(B)**	-3.32	-2.95	-3.73	-3.79	-9.39	-3.41	-0.81	-3.77	-4.91	-3.45	-3.25	-10.78	-10.55

Table S3. Electrostatic (*ele*) interactions (in Kcal/mol) computed for all TYR systems.

Residue	6a	6b	6c	6e	6f	6h	6i	6j	6l	6m	6n	6o	6p
**Phe197**	-0.03	0.18	0.01	0.06	0.03	0.17	0.14	-0.14	0.06	-0.15	0.27	-0.10	-0.01
**Gly200**	-0.30	0.01	-0.16	0.02	0.06	0.37	0.20	-2.38	-0.33	-0.49	-0.46	-0.28	-0.24
**Pro201**	-0.18	-0.02	0.01	-0.20	-0.05	0.06	-0.03	-0.36	-0.12	-0.30	-0.31	-0.18	-0.22
**His204**	2.50	2.30	2.42	2.38	2.25	2.39	1.21	2.05	2.36	1.99	2.35	2.36	2.19
**Asn205**	0.43	0.78	0.52	0.68	1.17	0.79	-0.50	0.20	0.30	0.09	0.30	0.57	0.34
**Arg209**	-1.53	-1.82	-1.96	-0.85	-1.81	-3.30	0.58	0.58	-2.63	0.58	-1.07	-4.94	-1.34
**Met215**	-0.29	-0.35	-0.49	-0.55	-0.05	-0.43	-0.26	-0.32	-0.39	-0.37	-0.01	-0.45	-0.26
**Gly216**	0.40	0.07	0.44	-0.36	-0.12	0.05	-0.47	0.05	-0.07	-0.45	0.01	0.04	-0.14
**Val217**	-0.30	-0.35	-0.60	-0.32	-0.42	-0.45	-0.46	-0.16	-0.49	-0.11	-0.06	-0.63	-0.52
**Val218**	-0.15	-0.04	-0.18	0.06	-0.26	-0.12	0.13	0.02	0.05	0.09	0.09	-0.15	-0.03
**His231**	1.90	2.21	2.04	1.96	2.49	2.12	0.89	2.03	2.00	2.19	2.18	2.01	2.10
**Cu2(A)**	-6.88	-3.91	-4.60	-4.43	-1.14	-3.15	-35.79	-6.53	-4.11	-5.41	-5.77	-1.12	-2.83
**Cu2(B)**	-33.66	-36.35	-34.89	-35.82	-34.66	-34.96	-5.39	-36.80	-34.84	-36.98	-37.18	-31.92	-34.32
